# Outcomes of robotic surgery for inflammatory bowel disease using the Medtronic Hugo™ Robotic-Assisted Surgical platform: a single center experience

**DOI:** 10.1007/s00384-024-04736-2

**Published:** 2024-10-10

**Authors:** Matteo Rottoli, Stefano Cardelli, Giacomo Calini, Ioana Diana Alexa, Tommaso Violante, Gilberto Poggioli

**Affiliations:** 1 Surgery of the Alimentary Tract, IRCCS Azienda Ospedaliero-Universitaria di Bologna, Bologna, Italy; 2 Department of Medical and Surgical Sciences, Alma Mater Studiorum - University of Bologna, Via Massarenti, 9, 40138 Bologna, Italy

**Keywords:** Inflammatory bowel disease, Robotic surgery, Robotic platform, Hugo RAS, Outcome

## Abstract

**Purpose:**

The aim of the study was to compare the perioperative outcomes of patients affected by inflammatory bowel disease (IBD) who underwent surgery performed through laparoscopy or using the Medtronic Hugo™ RAS.

**Methods:**

This is a retrospective study from a prospectively maintained database comparing laparoscopic vs. robotic-assisted surgery for IBD from 01/11/2017 to 15/04/2024. All procedures were performed by a single surgeon robotic-naïve with a large experience in laparoscopic surgery for IBD. The robotic procedures were performed using the Medtronic Hugo™ RAS platform. Outcomes were 30-day postoperative complications, operative time, conversion rate, intraoperative complications, length of hospital stay, and readmission rate.

**Results:**

Among 121 consecutive patients, 80 underwent laparoscopic (LG) and 41 robotic-assisted surgery (RG). Baseline, preoperative and disease-specific characteristics were comparable except for older age (50 [38–56] vs. 38 [28–54] years; *p* = 0.05) and higher albumin level (42 [40–44] vs. 40 [38–42] g/L, *p* = 0.006) in the RG. The intracorporeal anastomosis was more frequent in the RG (80% vs. 6%; *p* < 0.001) with longer operative time (240 vs. 205 min; *p* = 0.006), while the conversion rate was not different (5% vs. 10%, *p* = 0.49). Surgical procedure types were equally distributed between the two groups, and the rate of intra-abdominal septic complication (IASC) was comparable across the different procedures. Postoperative complications were similar, including the rate of IASC (5% vs. 5%, *p* = 1), postoperative ileus (5% vs. 7.5%, *p* = 0.71), bleeding (2% vs. 5%, *p* = 0.66), and Clavien-Dindo > 2 complications (7% vs. 6%; *p* = 1).

**Conclusion:**

IBD surgery performed using the Medtronic Hugo™ RAS is safe and feasible, with similar postoperative outcomes when compared to the laparoscopic approach.

## Introduction

The management of Inflammatory bowel disease (IBD) frequently requires surgery. National and international guidelines advocate minimally invasive surgery as the preferred approach both for Crohn’s disease (CD) [1, 2] and ulcerative colitis (UC) [3, 4], whenever feasible.

The use of robotic platforms in colorectal surgery has grown exponentially over the last years [5]. A few reports from referral centers showed that robotic surgery is also feasible in the treatment of IBD and is associated with comparable or even improved postoperative outcomes as compared to the laparoscopic approach [6–9].

Among the different existing platforms on the market, the DaVinci platform has dominated the field of robotic surgery, with numerous studies validating its effectiveness across various surgical disciplines. Its established market presence results from proven quality outcomes, robust training programs, and a wide range of instruments. The introduction of the Medtronic Hugo™ Robotic-Assisted Surgery (RAS) platform offers a new contender in this field, promising to enhance surgical outcomes through its unique technological advancements [10–12].

The Hugo™ RAS system has been successfully introduced in urology, gynecology, and general surgery over the last 2 years. This system has technical characteristics that could potentially improve the implementation of the platform in colorectal surgery. The Hugo™ RAS platform differentiates itself from the DaVinci system with several innovative features, including a higher modularity and flexibility, an enhanced surgeon presence, and a potential cost-effectiveness for colorectal procedures. These tools potentially streamline the range of colorectal surgeries, making the platform a valuable addition to the field.

However, there is still a lack in the literature regarding the feasibility and outcomes of the use of this novel platform in the surgical treatment of IBD patients, which often require complex colorectal procedures.

Therefore, the aim of the study was to compare the perioperative outcomes of IBD patients who underwent surgery performed through laparoscopy or using the Medtronic Hugo™ RAS.

## Materials and methods

Institutional review board approval was obtained (Comitato Etico Area Vasta Emilia Centro, Regione Emilia-Romagna, approval number 270/2024/Oss/AOUBo, approved on May 16, 2024). This was a retrospective study including consecutive adult patients who underwent minimally invasive (laparoscopic or robotic) surgery for IBD from 01/11/2017 to 15/04/2024 at our institution. The data was retrieved from a prospectively maintained database hosted on the Alma Mater Studiorum University of Bologna REDCap (Research Electronic Data Capture) platform [13]. The robotic series started in July 2023. All cases included were followed in a multidisciplinary IBD setting involving our institution’s surgeon, IBD gastroenterologist, and dedicated nurses. There was no preoperative selection of patients who underwent robotic surgery, as factors such as the operating room availability and the waiting list primarily determined the approach. Patients younger than 18 years old, without a histologic diagnosis of IBD, and who underwent oncologic or emergent surgery were excluded from the analysis. All procedures were performed by a single surgeon with extensive experience in laparoscopic surgery for IBD (> 500 laparoscopic procedures) and who was robotic-naïve. The surgeon underwent the required certification for using the robotic platform and previously performed 20 other robotic procedures (colorectal non-IBD, cholecystectomies). Robotic procedures were performed using the Medtronic Hugo™ RAS platform (Medtronic, Minneapolis, MN, USA).

The primary outcome was the comparison of the 30-day overall postoperative complication rate. The secondary outcomes included the comparison of the operative time, conversion rate, intraoperative complications, length of hospital stay, and readmission rate.

The analyzed variables included demographics, age, sex, body mass index (BMI), smoking, American Society of Anesthesiologist (ASA) score, preoperative medical therapy (including steroid, immunomodulator or biologics), serum albumin level, hemoglobin, C-reactive protein, preoperative antibiotics, preoperative drainage of intra-abdominal collection, preoperative nutritional parenteral nutrition, previous abdominal surgery, indication for surgery, conversion (considered as a laparotomy created for any purpose other than specimen extraction) [14], associated surgical procedures, intracorporeal anastomosis (ICA) construction, intraoperative complications, operative time, and postoperative length of stay and readmission. ICA configuration was in all the cases a side-to-side isoperistaltic anastomosis performed with a Signia Tri-Stapling System. A V-Loc barbed suture was used for enterotomy closure. CD-specific characteristics, such as CD duration, CD age, CD behavior according to the Montreal classification, anoperineal disease, and complex CD (defined as the presence of an intraoperative intra-abdominal abscess, fistula, or inflammatory mass), were also collected. No modification has been made in the preoperative optimization between the two groups according to bowel preparation, medical therapy suspension, and preoperative sepsis treatment [15–18]. The postoperative complications were categorized according to the Clavien-Dindo classification [19]. The diagnosis of anastomotic leakage (AL) was based on the validated definition proposed by the International Study Group of Rectal Cancer (ISREC), which considered AL also the pelvic abscesses located in the proximity of the anastomosis, whether or not the origin was detectable [20]. Along with all types of postoperative intra-abdominal abscesses and entero-cutaneous fistula, they defined the intra-abdominal septic complication (IASC) category.

The study was conducted according to the Declaration of Helsinki guidelines, and the Ethical Committee approval was obtained. The manuscript was structured according to the STROBE cohort reporting guidelines [21]. Statistical analysis was conducted using R4.3.2 [22]. The continuous variables were summarized as median (IQR), and categorical variables were reported as frequency (percentage). Continuous variables were compared between the robotic group (RG) and laparoscopic group (LG) using the Wilcoxon rank-sum test, and categorical variables were compared using the chi-squared or Fisher’s exact test, as appropriate. Statistical significance was defined as a two-sided *p*-value < 0.05.

## Results

Between November 2017 and April 2024, 121 consecutive patients were included in the study. Of these, 80 were performed laparoscopically, and 41 underwent robotic-assisted surgery.

Table 1 summarizes the demographic data. Baseline characteristics were comparable between the LG and RG, except for age, since patients in the RG were older (mean age 50 vs. 38 years; *p* = 0.05).

**Table 1:** Demographics

	Robotic (tot = 41)	Laparoscopic (tot = 80)	*p*-value
Age, median [Q1–Q3]	50 [37.5–55.5]	38 [28–54.4]	0.05
Male gender	22 (56%)	48 (60%)	0.63
BMI, median [Q1–Q3]	23 [22–24]	20 [18.5–21.5]	0.14
Smoking history			0.16
Never smoked	13 (32%)	42 (52.5%)	
Suspended	9 (22%)	18 (22.5%)	
Smoking now	13 (32%)	17 (21%)	
ASA, median [Q1–Q3]	2 [2–2]	2 [2–2]	0.66
Previous abdominal surgeries	12 (29%)	13 (16.5%)	0.15
Preoperative therapy			
Antibiotics	3 (7%)	9 (11.5%)	0.74
Steroids	6 (15%)	19 (24%)	0.33
Immunomodulators	1 (2%)	3 (4%)	1
Previous biologic therapy	26 (63%)	38 (47.5%)	0.14
Current biologic therapy	0	2 (2.5%)	0.51
Multiple biologic therapies	19 (46%)	23 (29%)	0.44
Years of disease, median [Q1–Q3]	7 [3–14]	10 [7–13]	0.33

*BMI* body mass index, *ASA* American Society of Anaesthesiologists

The preoperative disease-specific characteristics were comparable between the two groups, as reported in Table 2, including the preoperative exposure to biologic therapies (46% vs. 50%; *p* = 1).

**Table 2:** Crohn’s disease-specific characteristics

	Robotic (tot = 21)	Laparoscopic (tot = 42)	*p*-value
Penetrating disease behavior	6 (29%)	18 (43%)	0.35
Recurrent CD	1 (5%)	0	0.32
Complex CD	4 (19%)	17 (40%)	0.12
Preoperative parenteral nutrition	0	4 (9%)	0.28
Preoperative biologic therapy	11 (46%)	21 (50%)	1
Current	0	2 (5%)	
Multiple previous lines	7 (34%)	12 (29%)	
Perianal CD requiring surgery	3 (14%)	6 (14%)	1
Ileal resections*	2 (9.5%)	2 (5%)	1
Ileocecal resection*	18 (86%)	31 (74%)	1
Intracorporeal anastomosis	16 (80%)	2 (6%)	< 0.001

*CD* Crohn’s disease

^*^Side-to-side stapled anastomosis

Ileocecal resection with a stapled side-to-side ileo-colic anastomosis was the most frequent surgical intervention (18/21 in the RG and 31/42 in the LG). Notably, ICA construction was more frequently performed with the robotic-assisted platform (80% vs. 6%; *p* < 0.001).

The intraoperative and postoperative surgical characteristics are summarized in Table 3. CD and UC diagnosis and type of procedures were similar between the two groups. Patients in RG had a higher median (IQR) albumin level as compared to the LG (42 g/L [40–44] vs. 40 g/L [38–42], *p* = 0.006).

**Table 3:** Perioperative and postoperative surgical characteristics

	Robotic (tot = 41)	Laparoscopic (tot = 80)	*p*-value
Albumin level at surgery (g/L), median [IQR]	42 [40–44]	40 [38–42]	0.006
Hemoglobin at surgery (g/L), median [IQR]	12.5 [11–13]	12 [11–13.5]	0.15
Open conversion	2 (5%)	8 (10%)	0.49
CD diagnosis	21 (51%)	42 (52.5%)	1
UC diagnosis	20 (49%)	38 (47.3%)	1
Surgical intervention type			0.59
Ileal or ileocecal resection alone	20 (49%)	32 (40%)	
Ileocecal resection and concomitant colonic resection	0	3 (4%)	
Right extended colonic resection	1 (2%)	7 (8.5%)	
Proctectomy and IPAA	16 (39%)	28 (35%)	
Abdominal perineal resection	2 (5%)	3 (4%)	
Total colectomy	2 (5%)	7 (8.5%)	
Duration of surgery (min), median [IQR]	240 [185–290]	205 [150–240]	0.002
Length of hospital stay (days), median [IQR]	6 [6–7]	6 [6–7]	0.78
Postoperative medical complication	4 (10%)	7 (8.5%)	0.74
Postoperative surgical complication	8 (20%)	14 (17.5%)	0.98
Intra-abdominal septic complications	2 (5%)	4 (5%)	1
Paralytic ileus	2 (5%)	6 (7.5%)	0.71
Bowel occlusion	1 (2%)	2 (2.5%)	1
Bleeding complication	1 (2%)	4 (5%)	0.66
Clavien-Dindo classification > 2	3 (7%)	5 (6%)	1
30-day mortality	0	0	–

*IQR* interquartile range, *CD* Crohn’s disease, *UC* ulcerative colitis, *IPAA* ileal pouch-anal anastomosis

The operative time was significantly longer in the RG group than in the LG group (240 vs. 205 min; *p* = 0.006), while the conversion rate was comparable (5% vs. 10%, *p* = 0.49).

Postoperative medical and surgical complications were similar between the two groups, including the rate of intra-abdominal septic complications (5% vs. 5%, *p* = 1), postoperative ileus (5% vs. 7.5%, *p* = 0.71), and bleeding (2% vs. 5%, *p* = 0.66). The rate of Clavien-Dindo > 2 complications was also similar in the two groups (7% vs. 6%; *p* = 1). More specifically we reported the rate of IASC based on specific surgical procedures in Table 4. The three patients who had postop complications with a Clavien-Dindo score of 2 or more in the RG underwent an ileocecal resection and two restorative proctectomies with IPAA formation, while the four patients in the LG underwent an ileocecal resection for CD, a colonic resection for CD, a total colectomy for UC, and a restorative proctectomy with IPAA formation.

**Table 4:** Anastomotic leakage upon different surgical procedures

	No IASC robotic group (tot = 39)	IASC robotic group (tot = 2)	No IASC laparoscopic group (tot = 76)	IASC laparoscopic group (tot = 4)
Ileal or ileocecal resection	19 (49%)	1 (25%)	31 (41%)	1 (25%)
Ileocecal resection and colonic resection	0	0	3 (4%)	0
Colonic resection	1 (3%)	0	6 (8%)	1 (25%)
IPAA	15 (38%)	1 (25%)	28 (37%)	0
Abdominoperineal resection	2 (5%)	0	2 (3%)	1 (25%)
Total colectomy	2 (5%)	0	6 (8%)	1 (25%)

All *p*-values are not significant

*IASC* intrabdominal septic complication, *IPAA* ileal pouch-anal anastomosis

## Discussion

Considering the recent introduction of this platform in the market, this is the first comparative study to report outcomes after surgery for IBD using the Medtronic Hugo™ RAS. The comparison of the two groups showed similar postoperative outcomes and comparable preoperative and intraoperative characteristics, confirming that no preoperative selection based on the clinical presentation, the behavior of the disease, or the anticipated difficulty of surgery was carried out. The robotic procedures included a range of common operations performed in patients with IBD, including ileocecal resection, right extended colectomy, total colectomy, restorative proctectomy with ileal pouch-anal anastomosis (IPAA), and abdominal perineal resection (APR). Such variability highlights the platform’s flexibility in terms of its application in colorectal surgery, regardless of the case’s complexity.

The comparable rates of complication and conversion between laparoscopic and robotic surgery support the smoothness of the transition between the two approaches since the present surgeries were carried out by a robotic-naïve surgeon. In addition, the transition is made easier thanks to the similarity between laparoscopy and the Hugo™ RAS platform approach. This permits a freer trocar positioning, resulting in an intrabdominal surgical approach very similar to the already standardized laparoscopy, with the addition of the benefits of the robotic platform. Differences in operative time are a consequence of the starting approach, expected to decrease as the number of procedures performed increases and the learning curve progresses. Although defining the learning curve will require a larger, possibly multicentric series, [23] these early findings suggest that laparoscopic expertise might translate to the robotic approach yielding non-inferior outcomes using this platform.

Not surprisingly, a lower rate of ICAs was found in the laparoscopic group, and similar findings were shown in previous studies [24]. Laparoscopic suturing with intracorporeal knot tying or uncomfortable position with the need for additional trocars placement might represent, in fact, a limitation to the intracorporeal anastomotic construction. Moreover, the transection of the increasingly thickened mesentery of the ileum of CD patients often represents a challenge due to the tendency to bleed and the difficulty in obtaining a decent hemostasis, also using advanced energy devices. The advantages of the robotic platform in terms of 3D visualization, articulating instruments, improved precision, and the use of bipolar forceps (which are more effective in controlling mesenteric bleeding) likely led to greater confidence when performing the mesenteric division and the subsequent anastomosis, which often sits in uncomfortable positions as the ascending colon is usually preserved. More specifically, in our experience, the Hugo™ RAS platform’s bipolar forceps provide adequate hemostasis for many procedures, and only in selected cases, additional advanced energy devices such as Ligasure were employed for managing more complex mesenteric bleeding. ICA is associated with advantages including using off-midline extraction, better pain control, an earlier return to bowel function, and minimizing potential tension placed on thickened and foreshortened mesentery, leading to improved recovery and long-term outcomes [7–9, 25, 26].

The benefits of robotic-assisted proctectomy and IPAA reported in previous studies [27–31] were also confirmed in this early experience with the Medtronic Hugo™ RAS platform, particularly regarding the enhanced articulation in the low pelvis and the possibility of mobilizing the rectum distally to the level of the anorectal junction more easily in order to achieve a short rectal cuff.

The limitations of the present study included the retrospective nature, the single center and single surgeon design, and the small sample size. However, this early experience supports the extensive use of Medtronic Hugo™ RAS in colorectal and IBD surgery. The technical characteristics of the platform accommodate some of the needs of colorectal surgery, which are not found in other specialties, such as urology or gynecology. Being a modular system, in fact, it might overcome the intrinsic difficulties associated with the large surgical field of colorectal surgery just by moving one or two arms in different docking set-ups while moving from one surgical field to the other (i.e., from the splenic flexure to the pelvis).

In conclusion, IBD surgery performed using the Medtronic Hugo™ RAS is safe and feasible, with similar postoperative outcomes when compared to the laparoscopic approach. Randomized multicentre studies will be needed to assess the clinical advantages in larger series.

## Data Availability

The data that support the findings of this study are not openly available due to reasons of privacy and sensitivity and are available from the corresponding author upon reasonable request. Data are located in controlled access data storage at Alma Mater Studiorum—University of Bologna.
